# Evaluation of the TRIP13 level in breast cancer and insights into potential molecular pathways

**DOI:** 10.1111/jcmm.17278

**Published:** 2022-03-23

**Authors:** Jin Lan, Jingzhan Huang, Xinyi Tao, Yuan Gao, Longshan Zhang, Weiqiang Huang, Junjie Luo, Chuqin Liu, Yunyao Deng, Lixin Liu, Xiaolong Liu

**Affiliations:** ^1^ 572489 Department of General Surgery The Third Affiliated Hospital of Southern Medical University Guangzhou China; ^2^ Department of Radiation Oncology Nanfang Hospital Southern Medical University Guangzhou China

**Keywords:** breast cancer, cell proliferation, prognosis, therapeutic target, TRIP13

## Abstract

TRIP13 is a member of the large superfamily of the AAA + ATPase proteins and is associated with a variety of activities. Emerging evidence has shown that TRIP13 may serve as an oncogene. However, the function of TRIP13 in breast cancer (BC) has not yet been elucidated. Here, a variety of bioinformatic tools and laboratory experiments were combined to analyse the expression patterns, prognostic value and functional network of TRIP13 in BC. Multiple databases and immunohistochemistry (IHC) indicated a higher TRIP13 expression in BC tissue compared with normal tissue. TRIP13 was highly expressed in lung metastatic lesions compared with primary tumours in a 4T1 cell implantation BALB/c mouse model of BC. Kaplan–Meier plots also revealed that high TRIP13 expression correlated with poor survival in patients with BC. Furthermore, gene set enrichment analysis revealed that TRIP13 was primarily enriched in the signalling pathway of PI3K‐AKT‐mTOR. Suppressing TRIP13 could inhibit the expression of related genes, as well as the proliferation and migration of BC cell. Finally, 10 hub genes with a high score of connectivity were filtered from the protein–protein interaction (PPI) network, including MAD2L1, CDC20, CDC5L, CDK1, CCNA2, BUB1B, RAD51, SPO11, KIF11 and AURKB. Thus, TRIP13 may be a promising prognostic biomarker and an effective therapeutic target for BC.

## 1 INTRODUCTION

Breast cancer (BC) is the most common malignancy in women worldwide, the prevalence of which is increasing.^1^, ^2^ In China, BC had become the leading cause of mortality in females, even though the population is getting younger.^3^ It is estimated that 20%–30% of BC patients experience distant metastasis.^4^ Although recent advances in cancer therapy have been noteworthy, the underlying molecular mechanisms of progression in BC have not been fully elucidated. Therefore, the identification of novel therapeutic targets for BC is urgently required.

Thyroid hormone receptor interactor 13 (TRIP13, also known as pachytene checkpoint 2 and 16E1BP), was found to interact with human papillomavirus E1 proteins, also playing a key role in meiotic recombination, chromosome synapsis and spindle assembly checkpoint.^5^, ^6^ There is mounting evidence that TRIP13 protein levels are overexpressed in several human cancers, including ovarian cancer, colorectal cancer, prostate cancer and Wilms' tumour.^7^, ^8^, ^9^, ^10^ Aberrant expression of TRIP13 may be related to the occurrence and development of tumours, and the up‐regulation of TRIP13 promotes cell proliferation and migration and increases resistance to chemotherapeutic drugs.^9^, ^10^, ^11^ However, the function of TRIP13 in BC has not yet been elucidated.

In the present study, bioinformatics analysis integrated with laboratory experiments was used to determine whether TRIP13 acts as a novel oncogene in BC. Thus, multiple methods were performed to unravel the role that TRIP13 plays in the development of BC. The purpose of the study was to better understand the development of BC and to find novel targets for its therapy, thereby preventing the development of disease and its progression.

## 2 MATERIALS AND METHODS

### 2.1 Breast carcinoma datasets

The breast cancer gene expression profiles GSE29431 and GSE42568 were obtained from the publicly available Gene Expression Omnibus (GEO) database (http://www.ncbi.nlm.nih.gov/geo/). The GSE29431 dataset contained 66 samples, from 54 tumours and 12 normal tissues in BC patients. The GSE42568 dataset contained 17 normal tissues and 104 tumour tissues. In addition, gene expression profiles of 1218 BC patients and their clinical information were selected from the Cancer Genome Atlas (TCGA, http://tcga‐data.nci.nih.gov), from which matched clinical and expression information was sorted for additional analysis.

### 2.2 ONCOMINE database analysis

The levels of transcription of TRIP13 in BC were examined using the publicly accessible Oncomine database (https://www.oncomine.org),^12^ containing cancer microarray data. Fold changes were recorded. *p*‐Values <0.05 were considered significant when comparing cancer and normal tissue.

### 2.3 UALCAN analysis

The UALCAN database (http://ualcan.path.uab.edu) includes TCGA RNA‐seq datasets and clinical data from 31 cancer types, allowing analysis of the relationship between gene expression and clinical characteristics.^13^ In the present study, we evaluated the relationship between TRIP13 and patient clinical characteristics including cancer stage, pathological type and additional criteria recorded in the UALCAN database.

### 2.4 Prognostic survival analysis

The Kaplan–Meier plotter (http://kmplot.com/analysis) is a public online tool able to plot the impact of 54,675 genes on survival in a number of cancer types, including breast cancer, ovarian cancer, liver and gastric cancer.^14^ Prognostic value analysis, including overall survival (OS), relapse‐free survival (RFS), post‐progression survival (PPS) and distant metastasis‐free survival (DMFS) were calculated using the Kaplan–Meier method. A *p*‐value <0.05 was considered statistically significant.

### 2.5 LinkedOmics analysis (functional enrichment analysis)

The LinkedOmics database (http://www.linkedomics.org) is a web‐based data‐mining platform for the analysis of TCGA cancer datasets and was used to probe differentially expressed genes in association with TRIP13 in the TCGA BC cohort, presented here as volcano plots and heat maps.^15^ Correlation data results were signed, ranked and used to perform enrichment analysis of the Gene Ontology (GO) database. GO terms are categorized into one of three groups: biological process (BP), cellular component (CC) and molecular function (MF). For the rank criterion, a *p*‐value <0.01, FDR <0.25, and 1000 simulations were used in the calculation.

### 2.6 Gene set enrichment analysis

Gene set enrichment analysis (GSEA) can be used to identify the pathways of biological mechanisms according to the expression matrix. In order to probe downstream signalling pathways correlated with TRIP13, the GSE2034 dataset was divided into two groups based on median TRIP13 expression levels. GSEA software (v2.1.0, Broad Institute) was then used to identify gene sets with a high enrichment score for TRIP13. Enrichment results were considered significant where the *p*‐value <0.05 and FDR <0.25.

### 2.7 PPI network construction analysis

The String Database (https://string‐db.org) is an online public database from which insights into the functional associations between proteins can be determined.^16^, ^17^ The protein–protein interaction (PPI) network of TRIP13 was constructed using String. Settings with a combined score >0.7 were considered to have high confidence levels. The PPI network was then visualized using Cytoscape software (version 3.5.1).

### 2.8 Patients tissue specimens

Twenty paraffin‐embedded BC tissues and paired normal controls were obtained from the Department of Pathology, Third Affiliated Hospital of Southern Medical University. All paraffin‐embedded tissues were cut into 2.0 μm slices and transferred to glass slides for further use. Informed consent for the collection of specimens was obtained from each patient prior to surgery. The study was approved by the ethics committee of The Third Affiliated Hospital of Southern Medical University.

### 2.9 Cell lines and animal models

4T1 and MDA‐MB‐231 breast cancer cells were obtained from the American Type Culture Collection (ATCC) and preserved in the Key Laboratory of Molecular Tumour Pathology, Southern Medical University. 4T1 was cultured in RPMI 1640 culture medium (Gibco) supplemented with 10% foetal bovine serum (HyClone), and MDA‐MB‐231 was cultured in DMEM (Gibco) supplemented with 10% FBS at 37°C in a humidified atmosphere containing 5% CO_2_.

Six‐week‐old female BALB/c mice were purchased from the Animal Center of Southern Medical University, Guangzhou, China, and housed in a specific pathogen‐free environment. A total of 10 × 10^4^ 4T1 cells were injected into the mammary fat pad of each mouse. After 30 days, all mice were sacrificed and their lungs and primary tumours removed. The harvested organs were fixed in formalin, then embedded in paraffin until required for additional research. All study protocols for the mice were approved by the Institutional Animal Care and Use Committee of Southern Medical University.

### 2.10 Immunohistochemistry

Specimens were embedded in paraffin then cut into 2.0 μm slices for immunohistochemistry (IHC). IHC staining was performed in the tissue sections by the following protocol. Firstly, sections were stepwise dewaxed in a gradient of ethanol concentrations to water. To block endogenous peroxidase activity, the sections were immersed in hydrogen peroxide solution. The sections were then incubated with anti‐TRIP13 antibodies (Proteintech, 19602‐1‐AP, 1:100 dilution) overnight at 4°C and then with a secondary antibody for 1 h followed by DAB solution for 1 min at room temperature. The sections were counterstained with haematoxylin for 5 min, then washed three times in PBS for 5 min each. Stained tissue sections were evaluated using a light microscope. Results were recorded as the degree of staining intensity and percentage of positive tumour cells.

### 2.11 Western blot analysis

Proteins from tissues and cell lines were prepared by lysis of cells using RIPA buffer (KeyGEN BioTECH) and quantified using a Bradford protein assay (KeyGEN BioTECH). The lysates were separated using sodium dodecyl sulphate‐polyacrylamide gel electrophoresis (SDS‐PAGE), then transferred onto PVDF membranes (Millipore). PVDF membranes were incubated with anti‐TRIP13 antibody (Proteintech, 19602‐1‐AP, 1:1000 dilution), GAPDH (Proteintech, 60004‐1‐Ig, 1:2000 dilution), PI3 Kinase, Phospho‐PI3K, AKT, Phospho‐ AKT, Phospho‐mTOR, Phospho‐p70 S6 Kinase, Phospho‐4E‐BP1 (diluted 1:1000, Cell Signaling Technology) overnight at 4°C, then with a specific HRP‐conjugated antibody (Fdbio Science, FDM007 or FDR007, 1:10,000 dilution). Immunoreactive protein signals were detected using FDbio‐Femto ECL Western blotting detection reagents (Fdbio Science).

### 2.12 Establishment of TRIP13 knockdown cell line

The shRNAs of TRIP13 were purchased from RuiBiotech Company and transfected into MDA‐MB‐231 cell to establish the TRIP13 knockdown cell line. The sequences of the shRNAs are listed below:

shRNA#1 forward: 5′‐CCGGGCTACTCAACAGACATAATATCTCGAGATATTATGTCTGTTGAGTAGCTTTTTG‐3′

shRNA#1 reverse: 5′‐AATTCAAAAAGCTACTCAACAGACATAATATCTCGAGATATTATGTCTGTTGAGTAGC‐3′

shRNA#2 forward: 5′‐CCGGCACTTCTAACATCACCGAGAACTCGAGTTCTCGGTGATGTTAGAAGTGTTTTTG‐3′

shRNA#2 reverse: 5′‐AATTCAAAAACACTTCTAACATCACCGAGAACTCGAGTTCTCGGTGATGTTAGAAGTG‐3′

Western blot was used to evaluate the transfection efficiency.

### 2.13 Cell proliferation and migration assay

Colony formation assay was used for cell proliferation. 800 TRIP13 knockdown and control breast cancer cells were seeded into six‐well plates and supplemented with complete medium containing 10% FBS and cultured for 6 days until the visible cell colonies formed. After fixation by 4% paraformaldehyde, colonies were stained with 0.5% crystal violet and methanol for 20 min. For cell migration assay, 1 × 10^5^ cells were seeded into Transwell chambers (8‐μm pore; BD Falcon) under the serum‐free conditions and then complete medium with 10% FBS was added to the bottom chamber. After 36 h of incubation, the cells were fixed and stained as described previously. For wound healing assay, cells were seeded into six‐well plates respectively. By the use of a sterile 200‐μl pipette tip, a wound was mechanically scratched, and photomicrographs were taken at 0 h and at 72 h with microscope.

### 2.14 Statistical analysis

Statistical analyses, except where specified otherwise, were performed using web resources. A two‐tailed Student's *t*‐test and chi‐square test were conducted for comparison of differences between groups using SPSS v22.0 software. Survival relative to TRIP13 expression in BC patients was analysed using Kaplan–Meier plots. The receiver operating characteristic (ROC) analysis was performed to assess the sensitivity and specificity of TRIP13. The ROC curve was plotted by SPSS, and the area under the ROC curve (AUC) was used for the predicted value. *p*‐Values <0.05 were considered statistically significant (*p‐value <0.05; **p‐value <0.01; ***p‐value <0.001).

## 3 RESULTS

### 3.1 TRIP13 is overexpressed in breast cancer

**FIGURE 1:**
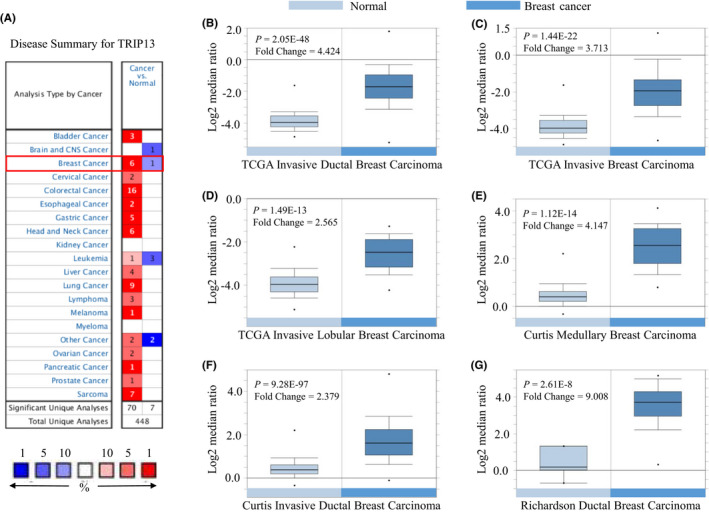
TRIP13 transcription in breast carcinoma (Oncomine). (A) mRNA expression of TRIP13 in different tumours. Graphs show the number of datasets with statistically significant mRNA overexpression (red) or down‐regulation (blue) of the target gene (cancer vs. normal tissue and cancer vs. cancer). *p*‐Value thresholds were 0.01. (B–G) Boxplots showing TRIP13 mRNA levels in sub‐database derived from Oncomine

**FIGURE 2:**
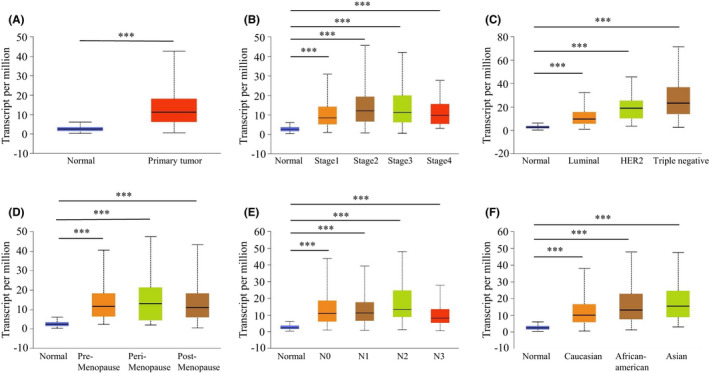
TRIP13 transcription in subgroups of patients with breast carcinoma, stratified based on stage, pathological type and other criteria (UALCAN). (A) Boxplot showing relative expression of TRIP13 in normal and breast cancer (BC) samples. (B) Boxplot showing relative expression of TRIP13 in normal individuals or BC patients in stages 1, 2, 3 or 4. (C) Boxplot showing relative expression of TRIP13 in normal individuals or BC patients of luminal, HER2 positive or triple negative. (D) Boxplot showing relative expression of TRIP13 in normal individuals of any age or in BC patients of pre‐menopause, peri‐menopause and post‐menopause. (E) Boxplot showing relative expression of TRIP13 in normal individuals or in BC patients in lymph node stages of N1, N2 or N3. (F) Boxplot showing relative expression of TRIP13 in normal individuals of any ethnicity or in BC patients of Caucasian, African‐American or Asian ethnicity. Data are mean ± SE. *p‐Value <0.05; **p‐value <0.01; ***p‐value <0.001

Transcription levels of TRIP13 in tumours were initially evaluated using data from the Oncomine database, revealing that TRIP13 was highly expressed in multiple tumours compared with normal samples, including BC (Figure 1A). Details of the analysis of 6 sub‐datasets indicated high transcriptional levels of TRIP13 in BC samples (Figure 1B–G and Table 1). Furthermore, UALCAN analysis indicated that the mRNA expression levels of TRIP13 were significantly higher in BC patients than in healthy controls using sub‐group analysis based on clinical stage, pathological features, menopause status, lymph node stage and ethnicity analysis (Figure 2). TRIP13 expression was highly correlated with tumour depth (*p* < 0.001) in BC patients (Table 2).

**TABLE 1:** Situation of each sub‐database

Database	Sample size	Sample size of normal	Sample size of BC	Fold change	*p*‐Value
TCGA breast	450	61	389	4.424	2.05E‐48
	137	61	76	3.713	1.44E‐22
	97	61	36	2.565	1.49E‐13
Curtis breast	176	144	32	4.147	1.12E‐14
	1700	144	1556	2.379	9.28E‐97
Richardson breast	47	7	40	9.008	2.61E‐8

Abbreviations: BC, breast cancer; TCGA, the Cancer Genome Atlas.

**TABLE 2:** Clinical features of BC patients with differential expression of TRIP13 in TCGA

	TRIP13 expression (%)		*p*‐Value
	Low (*n* = 389)	High (*n* = 389)	
Age			
<60	192 (45.7)	228 (54.3)	*p* = 0.01
>60	197 (55.0)	161 (45.0)	
Tumour depth			
T1	136 (66.0)	70 (34.0)	*p* < 0.001
T2	201 (43.4)	262 (56.6)	
T3	36 (45.6)	43 (54.4)	
T4	16 (53.3)	14 (46.7)	
Lymph node			
−	189 (50.1)	188 (49.9)	*p* = 0.943
+	200 (49.9)	201 (50.1)	
Distant metastasis			
−	380 (49.7)	384 (50.3)	*p* = 0.281
+	9 (64.3)	5 (35.7)	

Abbreviations: BC, breast cancer; TCGA, the Cancer Genome Atlas.

**FIGURE 3:**
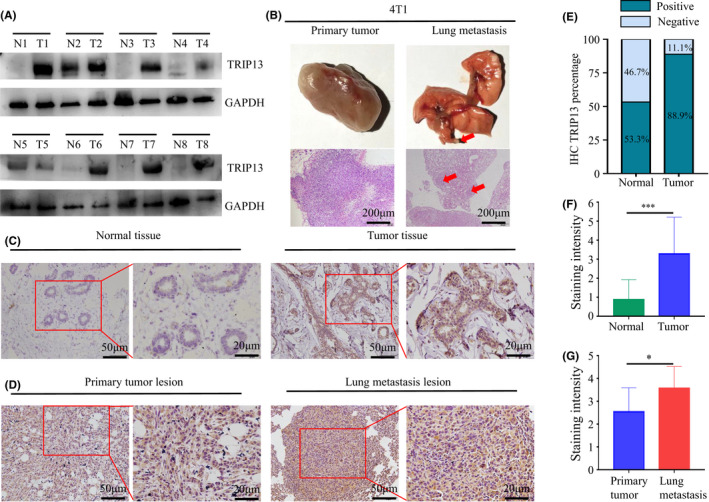
TRIP13 is overexpressed in breast cancer (BC) samples, especially in lung metastasis lesion. (A) Western blot analysis of TRIP13 in 8 paired BC tissues and corresponding normal tissues. (B) Images of primary tumour and lung metastasis lesion of mice injected with 4T1 and histopathological diagnosis (H & E staining) of samples from primary lesions and lung metastasis lesions. (C) TRIP13 in adjacent histologically normal tissue and tumour tissue of BC patients. (D) TRIP13 in primary lesions and lung metastasis lesion of BALB/c mice 4T1 BC models. (E) Percentage of TRIP13 IHC in BC and matched adjacent normal tissue. (F–G) IHC staining intensity of TRIP13 is shown. The IHC scale bars represent 50 µm and 20 µm. The HE scale bars represent 200 µm, respectively. *p‐Value <0.05; ** *p*‐value <0.01; *** *p*‐value <0.001

To verify the expression of TRIP13 in BC, Western blot analysis of 8 BC tumour samples and paired normal tissue were performed. In addition, 20 BC tumour samples and paired normal tissue were obtained and assessed using IHC staining. The results of Western blot and IHC analyses both indicated that TRIP13 is expressed at higher expression levels in BC tissues (Figure 3A,C). Moreover, the proportion of TRIP13 high expression in BC tissue is greater than that in normal tissues (Figure 3E), confirmed by the quantification of IHC staining (*p* < 0.001) (Figure 3F).

### 3.2 TRIP13 expression is higher in lung metastasis lesion in BC than that in primary lesions

In order to ascertain the role of TRIP13 in BC, an animal model of breast cancer was established, with histological diagnosis of primary tumour and lung metastatic lesion conducted using HE staining (Figure 3B). Interestingly, in the six 4T1 BC BALB/c model mice, TRIP13 expression was higher in lung metastatic tissue samples than in primary lesions (Figure 3D,G). This suggests that TRIP13 may play an important role in promoting BC metastasis.

### 3.3 High TRIP13 expression is associated with poor survival

**FIGURE 4:**
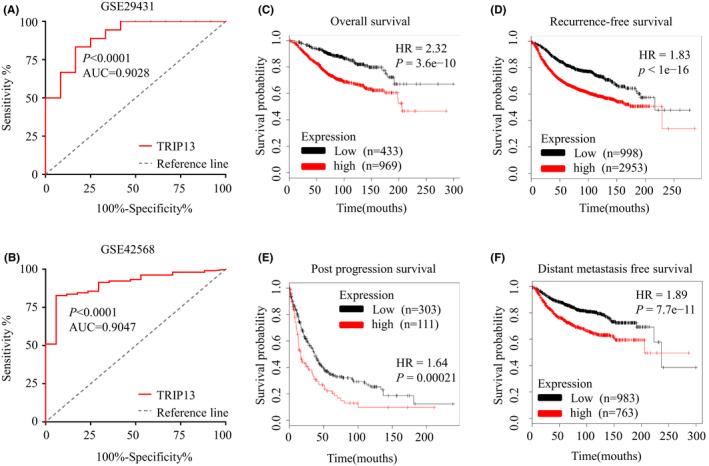
Diagnostic and prognostic value of TRIP13. (A)The AUC of TRIP13 for diagnosing breast cancer (BC) in GSE29431 was 0.9028 (*p* < 0.001). (B)The AUC of TRIP13 for diagnosing BC in GSE42568 was 0.9047 (*p* < 0.001). (C) High mRNA levels of TRIP13 were associated with shorter overall survival (OS) in BC patients. (D) High mRNA levels of TRIP13 were associated with shorter relapse‐free survival (RFS) in BC patients. (E) High mRNA levels of TRIP13 were associated with shorter PPS in BC patients. (F) High mRNA levels of TRIP13 were associated with shorter distant metastasis‐free survival (DMFS) in BC patients

To further evaluate the prognostic value of TRIP13 in BC patients, Kaplan–Meier plot survival analysis was performed. Survival analysis demonstrated that higher levels of TRIP13 expression were significantly associated with shorter OS and RFS in BC (Figure 4C,D). Similarly, high TRIP13 mRNA expression was also associated with decreased PPS and DMFS (Figure 4E,F). These results indicate that TRIP13 may represent a novel prognostic marker for BC.

In order to access the potential for TRIP13 to be used in the diagnosis of breast cancer, receiver operating characteristic (ROC) curve analysis was performed and area under the curve (AUC) values were calculated. As shown in Figure 4A and B, the AUC of TRIP13 for the diagnosis of BC in GSE29431 was 0.9028, and 0.9047 in GSE42568. The sensitivity and specificity of TRIP13 are applicable for the diagnosis of BC (greater than 0.7). Thus, TRIP13 expression could represent a potential diagnostic indicator for BC.

### 3.4 GO analysis of TRIP13 functional annotations in BC

**FIGURE 5:**
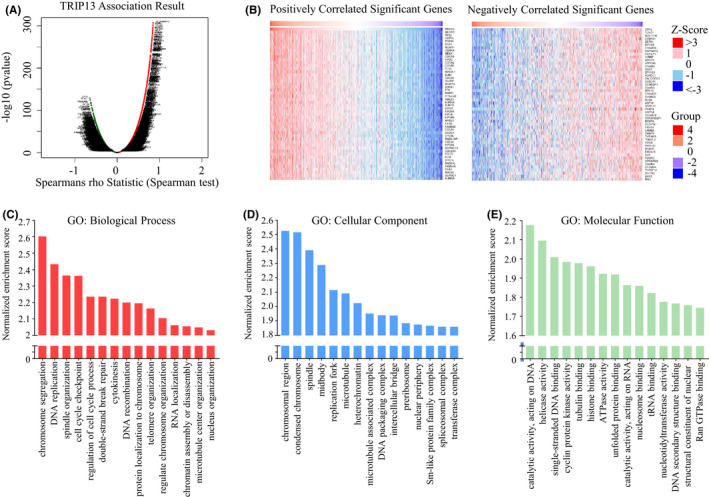
TRIP13 co‐expression genes in breast cancer (BC) (LinkedOmics) and GO annotations. (A) The global TRIP13 highly correlated genes identified by Pearson test in BC cohort. (B) Heat maps showing top 50 genes positively and negatively correlated with TRIP13 in BC. Red indicates positively correlated genes and blue indicates negatively correlated genes. (C–E) The top 15 GO annotations of TRIP13 in BC, including biological process (C), cellular component (D) and molecular function (E)

The functional enrichment tool of LinkedOmics was used to analyse mRNA microarray data from BC patients in the database from TCGA. As can be seen in the volcano plot (Figure 5A), 6179 genes were significantly positively correlated with TRIP13, while 7543 genes displayed a significant negative correlation. The 50 genes most significantly positively and negatively correlated with TRIP13 are presented in a heat map (Figure 5B). GO analysis demonstrated that the gene set expression correlated with TRIP13 in BC was primarily involved with chromosome segregation, DNA replication and spindle organization in the biological process category (Figure 5C). For the cellular component category, the chromosomal region was the most significantly enriched term (Figure 5D). Furthermore, a number of molecular function terms, such as catalytic activity, helicase activity and DNA binding were significantly enriched (Figure 5E). These findings indicate the basic and specific functions of TRIP13 in BC.

### 3.5 Functional and pathway enrichment of TRIP13 in BC

**FIGURE 6:**
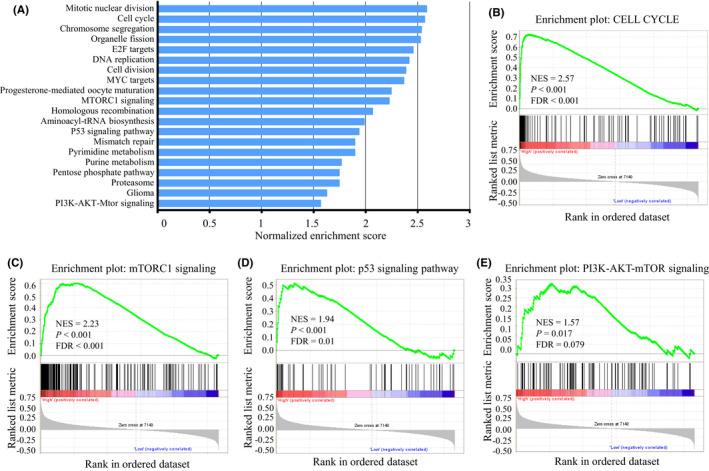
Gene set enrichment analysis reveals potential downstream signalling of TRIP13. (A) the top 20 positively enriched gene sets related to TRIP13. (B) Gene set enrichment analysis (GSEA) plots of cell cycle signalling. (C) GSEA plots of MTORC1 signalling. (D) GSEA plots of p53 signalling pathway. (E) GSEA plots of PI3K‐AKT‐mTOR signalling. NES, normalized enrichment score. Bottom panels show the ranking metrics of each gene. *Y*‐axis: ranking metric values; *X*‐axis: ranks for all genes

To further explore the potential mechanisms of TRIP13 in BC, GSEA was performed to compare the gene microarray profiles of TRIP13 in BC samples. The GSE2034 dataset contains 286 BC tissues divided into two groups based on their median TRIP13 expression levels: TRIP13^low^ (*n* = 143) and TRIP13^high^ (*n* = 143). The top 20 positively enriched gene sets related to TRIP13 expression in BC are presented in the blue column of the histogram (Figure 6A and Table 3). GSEA analysis indicated that the most significant functional pathways associated with TRIP13 were mitotic nuclear division, cell cycle and chromosomal segregation, participating primarily in the DNA replication and cell proliferation processes (Figure 6B). In addition, the mTORC1, p53 and PI3K‐AKT‐mTOR signalling pathways may also suggest that TRIP13 has metastatic activity (Figure 6C–E).

**TABLE 3:** Gene set enrichment analysis of co‐expressed genes with TRIP13 in breast cancer (BC)

Description	Count in gene set	NES	*p*‐Value
Mitotic nuclear division	205	2.59	*p* < 0.001
Cell cycle	105	2.57	*p* < 0.001
Chromosome segregation	182	2.54	*p* < 0.001
Organelle fission	297	2.53	*p* < 0.001
E2F targets	163	2.46	*p* < 0.001
DNA replication	100	2.42	*p* < 0.001
Cell division	399	2.39	*p* < 0.001
MYC targets	167	2.37	*p* < 0.001
Progesterone‐mediated oocyte maturation	74	2.25	*p* < 0.001
MTORC1 signalling	179	2.23	*p* < 0.001
Homologous recombination	23	2.07	*p* < 0.001
Aminoacyl‐tRNA biosynthesis	26	1.99	*p* = 0.002
P53 signalling pathway	58	1.94	*p* < 0.001
Mismatch repair	22	1.90	*p* = 0.004
Pyrimidine metabolism	72	1.90	*p* < 0.001
Purine metabolism	124	1.77	*p* < 0.001
Pentose phosphate pathway	23	1.75	*p* = 0.01
Proteasome	40	1.75	*p* = 0.034
Glioma	61	1.63	*p* = 0.006
PI3K‐AKT‐Mtor signalling	95	1.57	*p* = 0.017

Abbreviations: BC, breast cancer; NES, normalized enrichment score.

### 3.6 TRIP13 promotes BC cell proliferation and migration via the PI3K‐AKT‐mTOR pathway

**FIGURE 7:**
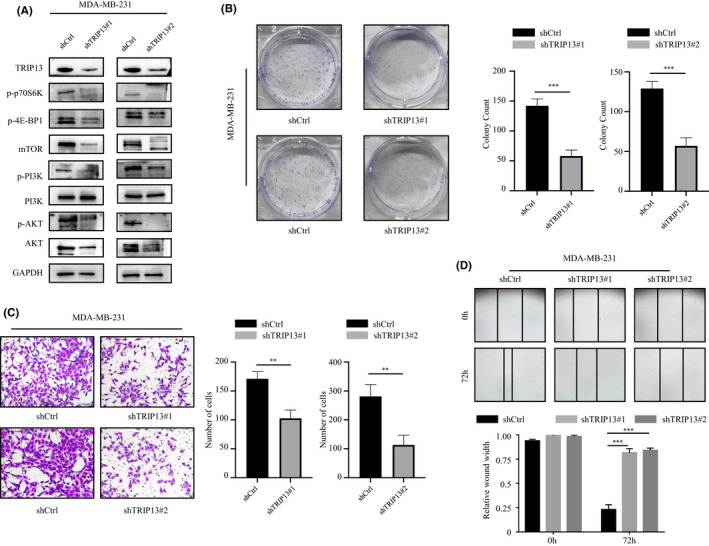
Function in proliferation and migration of TRIP13 and its regulated change of AKT pathway in breast cancer (BC) cell. (A) Western blot analysis showed changing of PI3K‐AKT‐mTOR signalling pathway in BC cell. (B) Effects of TRIP13 knockdown on BC cell proliferation were determined by colony formation assay. (C) Effects of TRIP13 knockdown on BC cell migration were determined by Transwell assay. (D) Effects of TRIP13 knockdown on BC cell migration were determined by Wound healing assay. *p‐Value <0.05; ** *p*‐value <0.01; *** *p*‐value <0.001

To explore the function of TRIP13 in BC progression, we checked the expression level of TRIP13 in MDA‐MB‐231 BC cell firstly. Then, we constructed plasmids with different shRNA sequences and individually transfected them into BC cells. The transfection efficiency was confirmed by Western blot (Figure 7A). Results of colony formation assay exhibited that TRIP13 knockdown reduced proliferative abilities of MDA‐MB‐231 cell (*p* < 0.01, Figure 7B). Transwell membrane assays and wound healing assays showed that TRIP13 knockdown greatly reduced the migratory abilities of BC cells (all *p* < 0.01, Figure 7C,D). These results suggest that TRIP13 is a metastasis promotor in BC.

Having confirmed the oncogenic role of TRIP13 in BC, we start to verify the molecular mechanisms. As GSEA predicted that TRIP13 may facilitate breast cancer progression through PI3K‐AKT‐mTOR signalling pathways. We performed Western blot to detect the expression of related proteins. As shown in Figure 7A, the activation of PI3K‐AKT‐mTOR signalling the expression of related markers such as p‐AKT, p‐PI3K, mTOR, p‐4E‐BP1 and p‐p70S6K was dramatically inhibited with silenced TRIP13. These results strongly suggest that TRIP13 triggers the activation of PI3K‐AKT‐mTOR signalling pathway.

### 3.7 PPI network and module analysis

**FIGURE 8:**
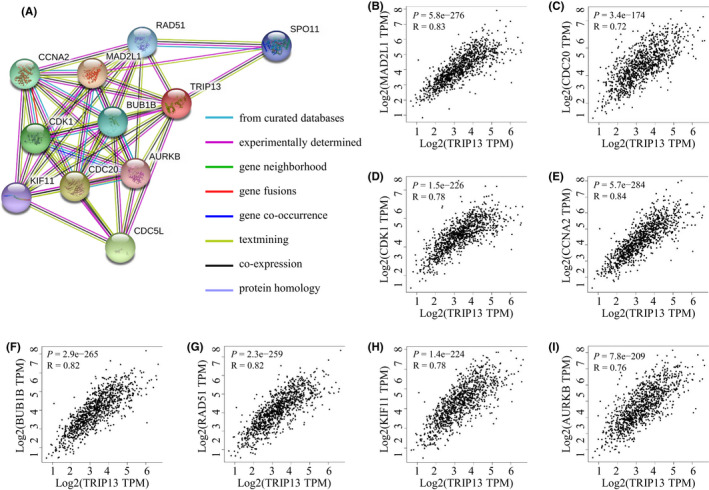
Protein–protein interaction (PPI) of TRIP13. (A)The network of TRIP13 and 10 proteins that significantly interacted with TRIP13 (String). (B–I) Pearson correlation coefficient plots between TRIP13 and hub genes (MAD2L1, CDC20, CDC5L, CDK1, CCNA2, BUB1B, RAD51, SPO11, KIF11 and AURKB)

Network construction identified 10 hub genes that significantly interacted with TRIP13, namely MAD2L1 (Mitotic Arrest Deficient 2 Like 1), CDC20 (Cell Division Cycle 20), CDC5L (Cell Division Cycle 5 Like), CDK1 (Cyclin Dependent Kinase 1e), CCNA2 (Cyclin A2), BUB1B (BUB1 Mitotic Checkpoint Serine/Threonine Kinase B), RAD51 (DNA Repair Protein RAD51 Homolog 1), SPO11 (Meiotic recombination protein SPO11), KIF11 (Kinesin Family Member 11) and AURKB (Aurora Kinase B) (Figure 8A). Pearson correlation coefficients were calculated by performing a correlation analysis of TRIP13 with hub genes (Figure 8B–I), which ranged from 0.72 (TRIP13 vs. CDC20) to 0.84 (TRIP13 vs. CCNA2). As with TRIP13, transcription levels of the hub genes were also elevated in BC (Figure S1). Expression of these hub genes often results in a poor prognosis (Figure S2).

## 4 DISCUSSION

TRIP13 is a member of the large AAA + ATPase protein superfamily. ATPases are involved in a variety of cellular activities, including protein degradation, DNA replication and chromosome synapsis.^6^, ^18^, ^19^ The majority of ATPases form hexamers to exert their biological function. Chemical energy generated by the hydrolysis of ATP in this protein family induces a conformational change in the substrate protein which modulates their functions.^20^, ^21^ Previous studies had shown that TRIP13 plays critical roles in meiotic recombination, DNA repair and spindle assembly checkpoint.^22^, ^23^ TRIP13 was first reported as an oncogene in head and neck cancer, demonstrating an ability to promote tumour growth and enhance the repair of DNA damage.^9^ Mounting evidence indicates that TRIP13 plays an oncogenic role in multiple human cancers, usually associated with poor survival.^9^, ^10^, ^24^, ^25^ Here, we explored the role of TRIP13 in BC.

From the analysis of BC datasets in the Oncomine and UALCAN databases, TRIP13 was found to be highly expressed in BC tumour tissue compared with normal controls (Figures 1 and 2), an observation validated by IHC analysis of physical samples from our BC patients (Figure 3A). Furthermore, the AUC values of TRIP13 expression in two gene expression profiles were greater than 0.7, suggesting that this characteristic was extremely relevant. In addition, high TRIP13 expression levels were significantly associated with poor prognosis (Figure 4C–F). Thus, together, these results suggest that TRIP13 overexpression occurs in BC and so represents a potential diagnostic and prognostic marker.

Surgery and radiation therapy are currently effective methods of controlling multiple cancers when they are primary lesions, but the development of metastatic tumours invariably results in unfavourable prognosis.^26^ Britta Weigelt and his colleagues noted that the metastatic capacity of a tumour is an inherent characteristic and not a necessarily late, acquired phenotype.^27^ Distant metastasis of tumour cells occurs early in many cancer patients. To explore the role of TRIP13 in BC metastasis, we established an animal model of BC. 4T1 murine mammary carcinoma cells are highly carcinogenic and reliably metastasize to multiple distant organs. The 4T1 mouse model, which to some extent resembles the formation of human breast cancers, allows the simulation of breast cancer metastasis progression in humans.^28^, ^29^ Interestingly, compared with primary BC lesions, TRIP13 expression is higher in lung metastatic lesions in our BALB/c mice 4T1 BC model (Figure 3C). Cancer cells from primary tumour manifest distinct gene expression patterns after metastasizing to distant organs. The interaction between tumour cells and extrinsic signals at metastatic organ critically affects the subsequent metastatic outgrowth.^30^, ^31^ Besides, the relationship between TRIP13 expression and the clinical features of BC patients in TCGA uncovered the correlation of TRIP13 expression with disease stage (Table 2). Our results in function assays confirmed that TRIP13 indeed promote tumour progression in BC. These results suggest that TRIP13 plays a critical role in metastasis during BC progression.

Since TRIP13 plays several critical physiological functions, its dysregulation may alter the transduction of various signalling pathways. To gain insight into the potential molecular functions of TRIP13 and its interactive network in BC, a multiple bioinformatics analysis of BC datasets was performed. Here, we present homologous changes in the gene transcriptome caused by TRIP13 expression through volcano plots and heat maps (Figure 5A,B). This variation substantially impacts the effect of TRIP13 on the BC transcriptome. Results of GO annotation analysis indicated that genes affected by TRIP13 were mostly enriched in chromosome segregation, DNA replication and spindle organization (Figure 5C–E). These annotations were principally related to cell proliferation and differentiation. Previous studies have demonstrated that TRIP13 promotes cell proliferation and invasion via interactions with 14‐3‐3 Theta (YWHAQ) and 14‐3‐3 Zeta (YWHAZ) in CRC.^10^ YWHAQ and YWHAZ are the members of the 14‐3‐3 superfamily which mediates G2‐M transition and epithelial‐mesenchymal transition (EMT).^32^ TRIP13 mutations predispose a cell to chromosome missegregation and tumorigenesis.^8^ These results suggest that elevated TRIP13 in BC cells may contribute to unnatural activation of these processes, leading to the development of BC.

Next, GSEA was also conducted to explore the potential molecular mechanisms of TRIP13‐driven BC progression and metastasis. The results indicate that TRIP13 expression was significantly associated with mitotic nuclear division, cell cycle progression, DNA replication and chromosomal segregation. The cell cycle process has four successive phases, each precisely regulated by checkpoints.^33^ During the evolution of cancer cell, this proofreading mechanism is abolished due to dysfunctional checkpoints. Thus, mismatched DNA can then be duplicated, leading to uncontrolled proliferation and malignant phenotype.^34^ We hypothesized that TRIP13 mediates the progression of BC through the regulation of tumour cell cycle signalling. In addition, the mTORC1, p53 and PI3K‐AKT‐mTOR signalling pathways often activated in diverse cancers were also involved in TRIP13‐related BC progression. Evidence has shown that mTOR‐related mechanisms contribute to malignant phenotypes in multiple cancers. mTORC1 promotes carcinogenesis by driving transcription of oncogenes and inducing angiogenesis. mTORC2 plays a role in the activation of AKT and other AGC family proteins which promote cell proliferation.^35^, ^36^ Loss of p53 and PI3K/AKT/mTOR activation promotes tumour development and metastasis.^37^, ^38^ Consistent with our expectations, TRIP13 knockdown efficiently repressed the expression of the p‐AKT, p‐PI3K, mTOR, p‐4E‐BP1 and p‐p70S6K, all of which are classic PI3K‐AKT‐mTOR signalling pathway related genes. These observations are consistent with the molecular pathways involved in BC tumorigenesis and metastasis, and critical to understanding how aberrations in TRIP13 can result in physiological dysfunction and even cancer, such as BC.

While investigating the regulators potentially responsible for TRIP13 dysregulation, 10 hub genes were filtered from the PPI network: MAD2L1, CDC20, CDC5L, CDK1, CCNA2, BUB1B, RAD51, SPO11, KIF11 and AURKB (Figure 8A). These hub genes may also similarly interact with each other via various signalling pathways in BC. Their physiological functions are primarily involved in cell cycle control and may act as a transcription activatior.^39^, ^40^ CDK1, CCNA2 and AURKB are the members of the serine/threonine protein kinase family. Progression through the cell cycle is driven by the cyclin‐dependent kinase (CDK) family and cyclins.^41^, ^42^, ^43^ Furthermore, the majority of hub genes are associated with poor overall survival (Figure S1). Drugs targeting the cell cycle‐regulatory proteins CDK4 and CDK6 have been approved for the treatment of breast cancer, and inhibitors targeting other CDKs are currently in clinical trials.^44^ These targets can be incorporated into the comprehensive treatment of BC. To gain more accurate correlation results, many additional experiments are required to verify the results in the present study and elucidate the molecular mechanisms.

In summary, the study provides multi‐level evidence for the value of TRIP13 and its potential as a novel therapeutic target in BC. Further studies are required to verify these hypotheses. Finally, these findings contribute to a better understanding of the role of TRIP13 in BC.

## 5 CONCLUSION

The expression patterns, diagnostic and prognostic values, functional enrichment, and PPI networks of TRIP13 in patients with BC were investigated in the present study. We demonstrated that TRIP13 is elevated in BC tissues, especially in lung metastatic lesions. TRIP13 can facilitate the proliferative and migratory abilities of BC cell. The study provides novel and promising insight into the regular networks and pathways of TRIP13 in BC. Taken together, these results suggest that TRIP13 promotes tumour development and may serve as a novel and potential diagnostic or therapeutic target in BC.

## CONFLICT OF INTEREST

All authors declare no conflict of interest.

## AUTHOR CONTRIBUTIONS

**Jin Lan:** Investigation (lead); Resources (lead); Software (lead); Writing – original draft (lead). **Jingzhan Huang:** Data curation (equal); Formal analysis (equal); Methodology (equal). **Xinyi Tao:** Resources (equal). **Yuan Gao:** Methodology (equal); Resources (equal). **Longshan Zhang:** Data curation (equal); Validation (equal). **Weiqiang Huang:** Validation (equal); Visualization (equal). **Junjie Luo:** Methodology (equal); Software (equal). **Chuqin Liu:** Visualization (equal). **Yunyao Deng:** Formal analysis (equal). **Lixin Liu:** Project administration (equal); Supervision (equal). **Xiaolong Liu:** Project administration (equal); Supervision (equal); Writing – review & editing (equal).

## Supporting information

Figure S1

Figure S2

## Data Availability

The datasets analysed in this study can be accessed in the Oncomine, UALCAN, LinkedOmics and STRING database. Further access to data is available from the corresponding author upon reasonable request.
