# Supplementary figures and images for: Evaluation of the TRIP13 level in breast cancer and insights into potential molecular pathways
Source: J Cell Mol Med. 2022 Mar 23;26(9):2673–85. doi: 10.1111/jcmm.17278 (PMC9077308; doi:10.1111/jcmm.17278)

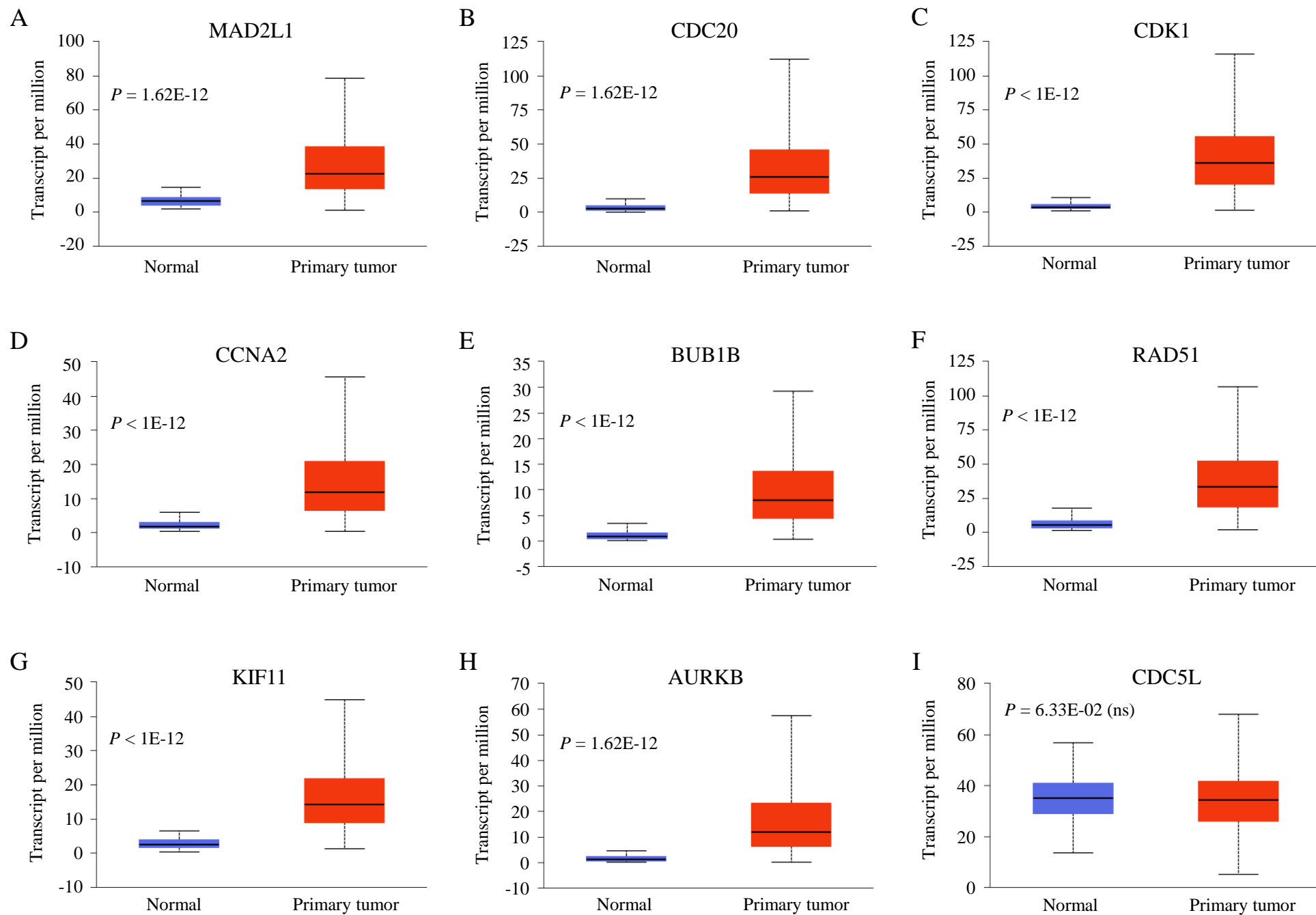

Supplement: Supplementary file 1 — Figure S1 [file JCMM-26-2673-s002.pdf]

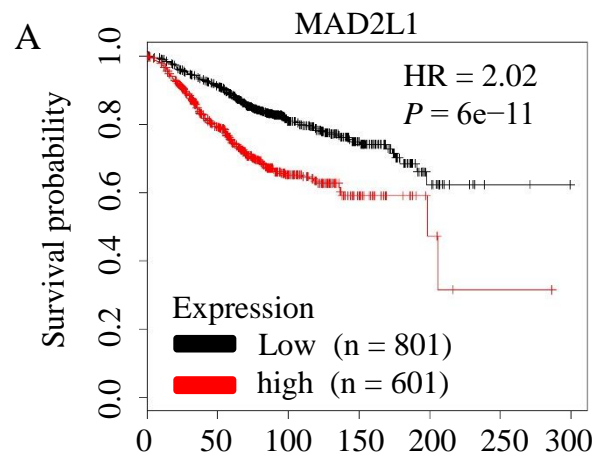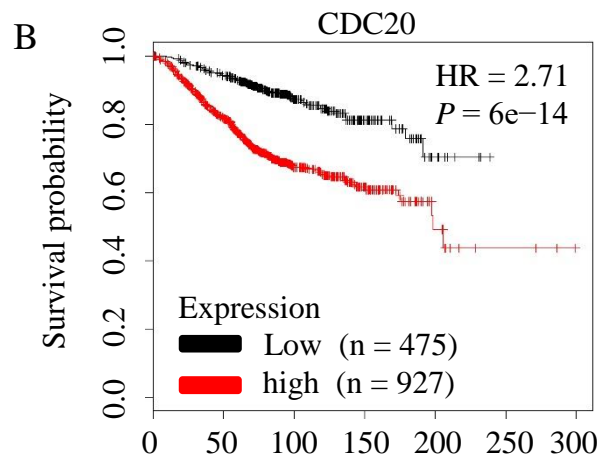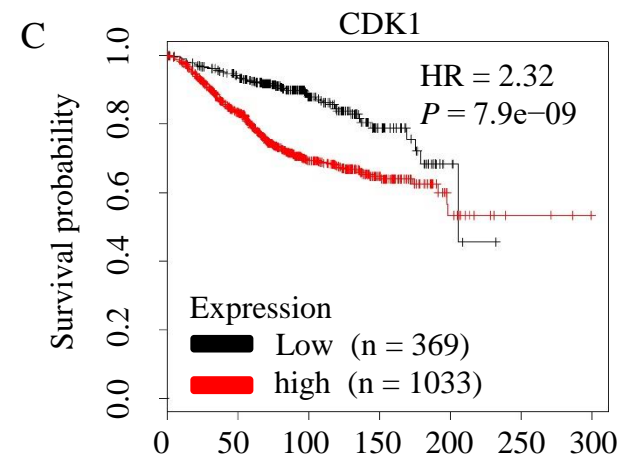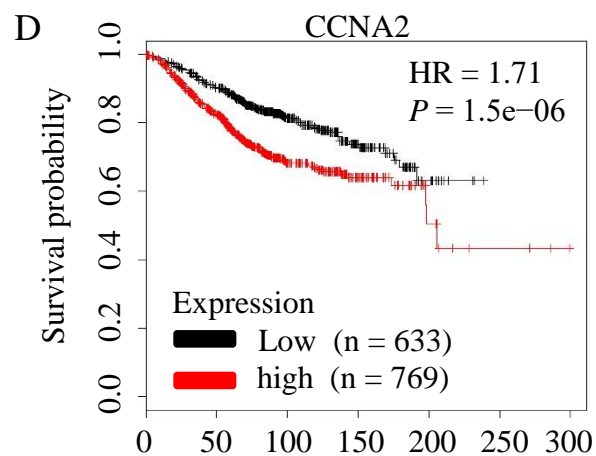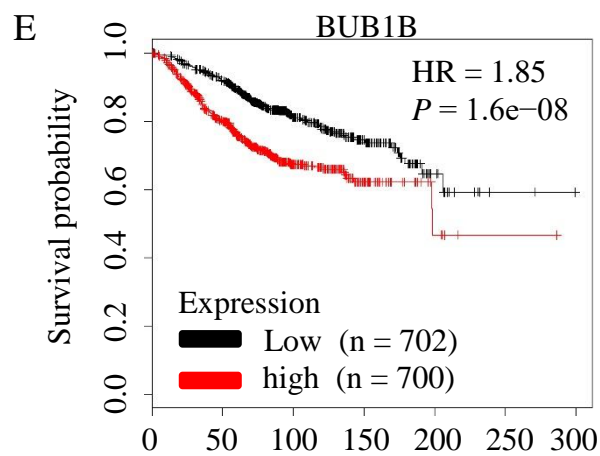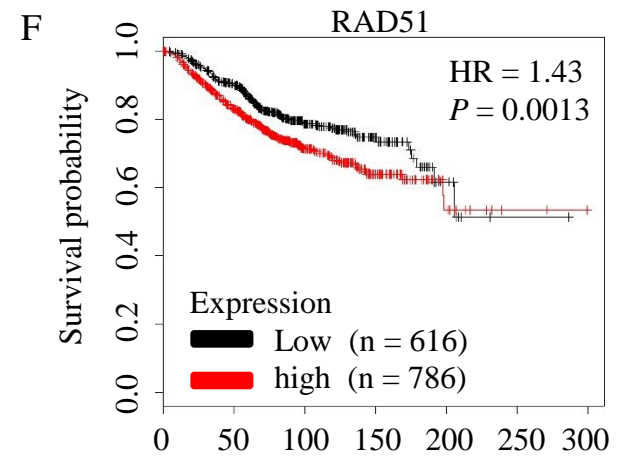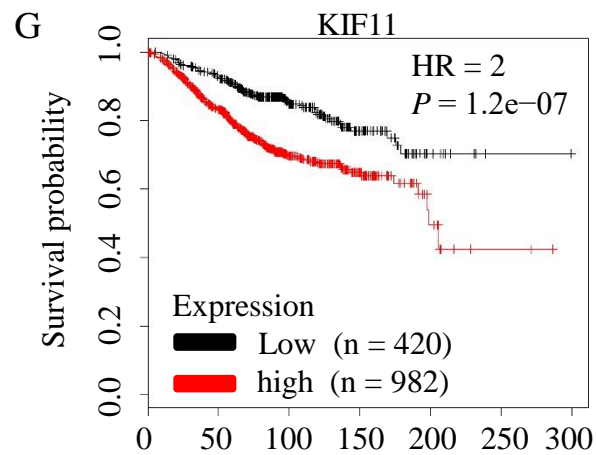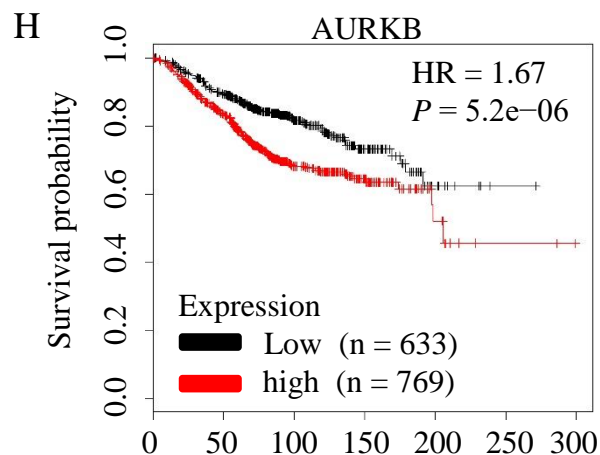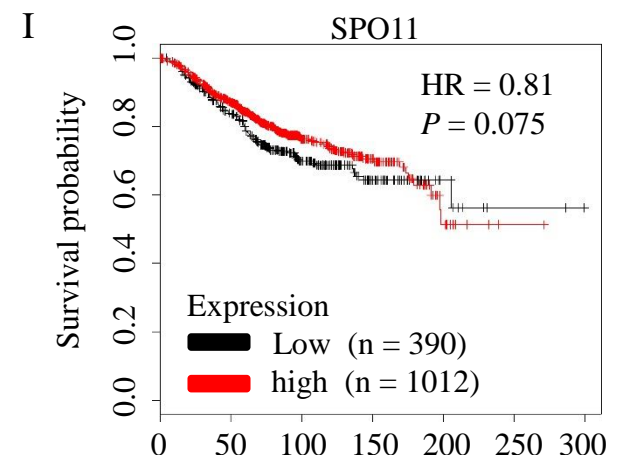

Supplement: Supplementary file 2 — Figure S2 [file JCMM-26-2673-s001.pdf]
